# Targeting strategies for oxaliplatin-induced peripheral neuropathy: clinical syndrome, molecular basis, and drug development

**DOI:** 10.1186/s13046-021-02141-z

**Published:** 2021-10-22

**Authors:** Yang Yang, Bing Zhao, Xuejiao Gao, Jinbing Sun, Juan Ye, Jun Li, Peng Cao

**Affiliations:** 1 Affiliated Hospital of Integrated Traditional Chinese and Western Medicine, Nanjing University of Chinese Medicine, 100#, Hongshan Road, Nanjing, 210028 Jiangsu China; 2 Department of Pharmacology, School of Pharmacy, Nanjing University of Chinese Medicine, Nanjing, 210023 China; 3 Jiangsu Key Laboratory for Pharmacology and Safety Evaluation of Chinese Materia Medica, Nanjing University of Chinese Medicine, Nanjing, 210023 China; 4 Yangtze River Pharmaceutical Group, Taizhou, 225321 China; 5 Changshu No.1 People’s Hospital Affiliated to Soochow University, Changshu, 215500 China; 6 Department of Pharmacy, The First Affiliated Hospital of Zhengzhou University, Zhengzhou, Henan 450052 P.R. China; 7 Zhenjiang Hospital of Chinese Traditional and Western Medicine, Zhenjiang, 212002, Jiangsu China

**Keywords:** Oxaliplatin-induced peripheral neuropathy, Clinical syndrome, Molecular basis, Drug development, Oxidative stress, Gut microbiota

## Abstract

Oxaliplatin (OHP)-induced peripheral neurotoxicity (OIPN) is a severe clinical problem and potentially permanent side effect of cancer treatment. For the management of OIPN, accurate diagnosis and understanding of significant risk factors including genetic vulnerability are essential to improve knowledge regarding the prevalence and incidence of OIPN as well as enhance strategies for the prevention and treatment of OIPN. The molecular mechanisms underlying OIPN are complex, with multi-targets and various cells causing neuropathy. Furthermore, mechanisms of OIPN can reinforce each other, and combination therapies may be required for effective management. However, despite intense investigation in preclinical and clinical studies, no preventive therapies have shown significant clinical efficacy, and the established treatment for painful OIPN is limited. Duloxetine is the only agent currently recommended by the American Society of Clinical Oncology. The present article summarizes the most recent advances in the field of studies on OIPN, the overview of the clinical syndrome, molecular basis, therapy development, and outlook of future drug candidates. Importantly, closer links between clinical pain management teams and oncology will advance the effectiveness of OIPN treatment, and the continued close collaboration between preclinical and clinical research will facilitate the development of novel prevention and treatments for OIPN.

## Background

**Fig. 1:**
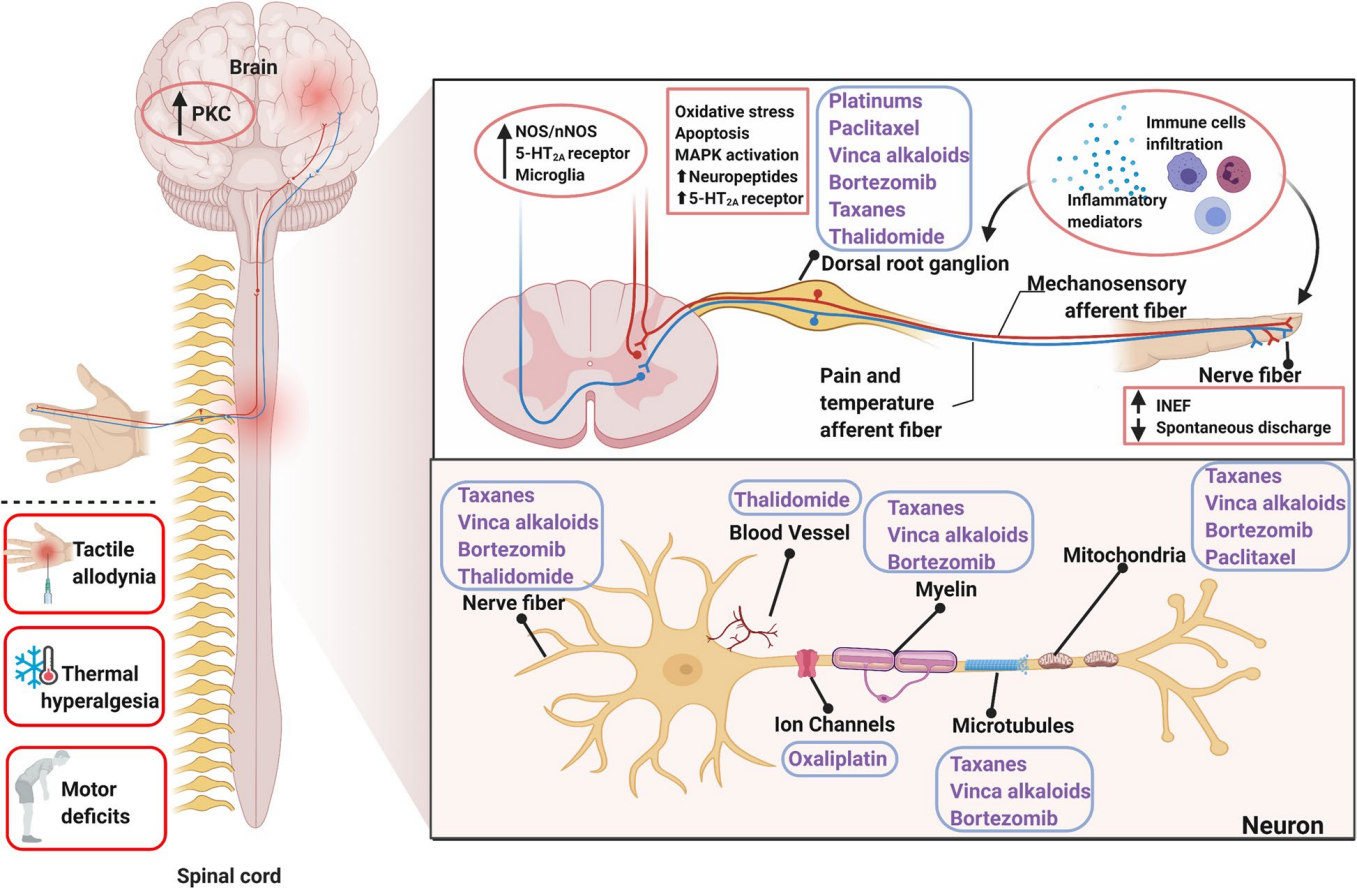
Sketch-map of the mechanism of chemotherapy-induced peripheral neuropathy (CIPN). Depiction of the typical symptoms and targets for CIPN toxicity in the peripheral nervous system depicted from the distal nerve terminals to axonal components (myelin, microtubules, mitochondria, ion channels, and vascular network), the dorsal root ganglion (DRG), and the central nervous system (CNS). CIPN was initiated and progressed by chemotherapeutic-agents through intraepidermal nerve fibers impairment, abnormal spontaneous discharge, activation of ion channels, up-regulation of neuro-immune system, oxidative stress, and the abnormal kinase activation in DRG and CNS. Contents in the blue boxes refer to different chemotherapy agents. Solid dots refer to the target of relative chemotherapeutic agents. Contents in the pink boxes refer to the pathological progress in peripheral and central nerve systems underlying CIPN

Chemotherapy-induced peripheral neuropathy (CIPN) is a serious clinical problem caused by cytotoxic drugs that cause different pathologic insults to neurons, including platinum, taxanes, proteasome inhibitors, vinca alkaloids, and immunomodulatory drugs with impairment progress in peripheral and central nerve systems, and presents in a “glove-and-stocking” symptom [1] (Fig. 1). Regarding overall neurotoxic chemotherapy, the prevalence of CIPN is approximately 68% at 1 month, reducing to 60% at 3 months and 30% at 6 months or more [2]. The type of chemotherapy influences the risk of developing CIPN and a wide range of occurrences are reported depending on different CIPN assessment and dosing regimens (Table 1).

**Table 1:** Chemotherapeutics and the incidence or prevalence of reported neuropathies

Chemotherapy	Class	Approximate incidence/prevalence of CIPN (%)	References
Oxaliplatin	Platinum-based chemotherapeutics	Acute: 85–96; chronic wide range: 40–93	[3]
Cisplatin		12–85	
Paclitaxel	Taxanes	61–92	[2, 4]
Bortezomib	Proteasome inhibitor	47–80	
Vincristine	Vinca alkaloids	14–70	[5]
Thalidomide	Immunomodulatory drugs	21–50	[6]

Data are mainly from randomized controlled trials or prospective cohort studies

*CIPN* Chemotherapy-induced peripheral neuropathy

Oxaliplatin (OHP) is the most prominent neurotoxic chemotherapy agent that interferes with tumor cell proliferation by forming DNA-platinum adducts, which lead to the destruction of cancer cells [7]. With the increasing clinical use of OHP, inevitably adverse reactions have been reported, with the major side effects being peripheral neurotoxicity, myelosuppression, and gastrointestinal reactions [8, 9]. These side effects of OHP may result in the terminations of treatment plans and a reduction in the compliance of colorectal cancer (CRC) patients during the treatment. This is important because OHP-induced peripheral neuropathy (OIPN) is the most common side effect associated with OHP dose-limiting toxicity [10]. In this paper, we reviewed the main progress of OIPN, focusing on the clinical syndrome, its molecular basis, and drug development.

## The clinical syndrome: classification and characteristics, clinical diagnosis, risk factors, and genetic polymorphisms with OIPN

### Classification and characteristics

**Fig. 2:**
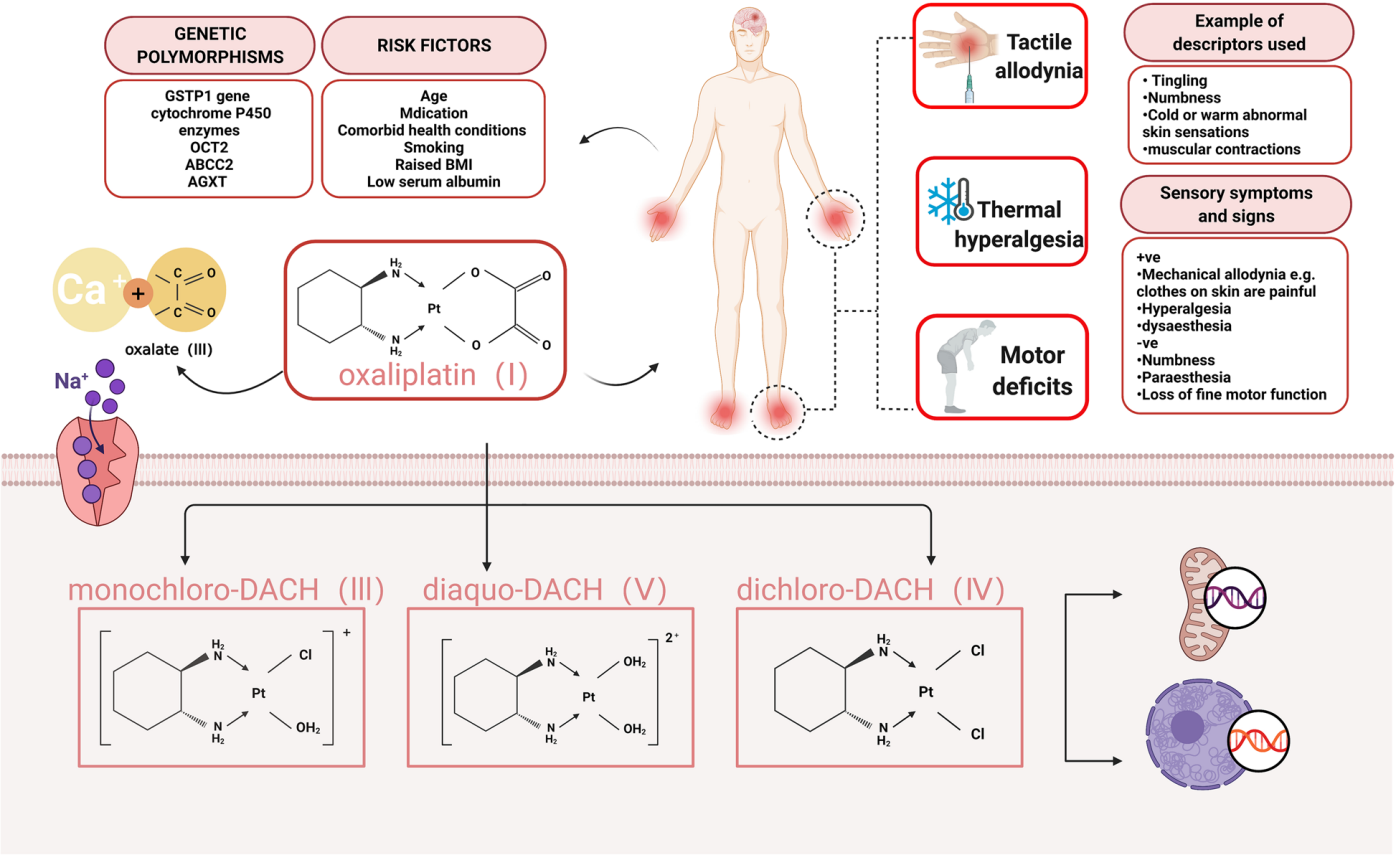
Oxaliplatin-induced peripheral neuropathy (OIPN)—Clinical features, risk factors, and main mechanism. OIPN is characterized by cold-sensitive peripheral paresthesia and motor symptoms. Risk factors (age, medication, comorbid health conditions, raised BMI, etc.) and genetic polymorphisms (GSTP1, OCT2, cytochrome P450, etc.) are associated with OIPN development. Chemical structure of oxaliplatin, its biotransformation pathways, and a potential mechanism underlying the development of oxaliplatin-induced neuropathy: oxaliplatin (I) is rapidly hydrolyzed in vivo to bioactive derivatives through the displacement of the oxalate group by H_2_O and Cl^−^ ions to produce oxalate (II) as well as reactive monochloro-diaminocyclohexane (DACH) (III), dichloroDACH (IV), and diaquo-DACH platinum (V) metabolites. Oxalate, which reacts with Ca^2+^ ions, causes transient impairment of the Na_v_ channel activation of the dorsal root ganglion (DRG) sensory neurons, and nerve hyperexcitability is the main contributor to neurotoxicity caused by oxaliplatin

OHP-induced neurotoxicity can be divided into two distinct forms, acute and chronic, according to the incidence, symptoms, duration, mechanisms, and other clinical features. Acute OIPN, triggered mainly by cold stimulation, occurs in 85–96% of patients within hours of infusion and lasts for the following 7 days [10, 11]. It is characterized by cold-sensitive peripheral paresthesia (hands and feet dysesthesia and paresthesia) and motor symptoms (e.g., prolonged muscular contractions, tetanic spasms, and fasciculations) [12] (Fig. 2). The most important mechanism involved in acute OIPN is the transient impairment of the Na_v_ channel activation of the dorsal root ganglion (DRG) sensory neurons and nerve hyperexcitability, due to oxalate metabolites [13, 14] (Fig. 2). Furthermore, the degree of acute neuropathy seems to predict the development of chronic neurotoxicity [7]. The development of chronic OIPN is likely to occur at cumulative doses exceeding 780–850 mg/m^2^ (40–93% incidence), and can even last for several years [7, 15–18]. Clinically, chronic OIPN may result in sensation loss and changes in proprioception that affect daily activities (Fig. 2). An important feature of chronic OIPN is the “coasting,” in which approximately 60% of patients report long-lasting neuropathic symptoms that significantly impair their quality of life after completing the last chemotherapy course [11, 19]. This is an important factor in deciding termination of treatment because of the neurotic symptoms, which can lead to disability [20]. The main mechanism responsible for the observed permanent distal sensory loss is associated with mitochondrial damage, the death of sensory neurons (i.e., nerve cell necrosis), glia activation, and neuroinflammation [21] (Table 2).

**Table 2:** Characteristics of acute and chronic OIPN

Characteristics	Acute OIPN	Chronic OIPN	References
Incidence rate	85–96%	40–93%	[7, 10, 11, 15–18]
Duration	Within hours of infusion and lasting for the following 7 days	Within 6–12 months, or even lasting for 5 years	
Typical feature	Cold-sensitive peripheral paresthesia, motor symptoms	Acute OIPN symptoms and the “coasting” phenotype	[7, 11, 19]
Mechanism	Na_v_ channel activation	Sensory neurons death, mitochondrial damage, oxidative stress, glia activation, and neuroinflammation, etc.	[13, 14, 21]

*OIPN* Oxaliplatin-induced peripheral neuropathy

### Clinical diagnosis

Accurate assessment and diagnosis are important in the management and understanding of the epidemiology of OIPN. However, there is currently no widely accepted, standardized assessment approach for the diagnosis of CIPN per se. A preliminary diagnosis of OIPN can be made according to a combination of signs and clinical symptoms, quantitative sensory tests (QST), and electrophysiological measurements (EPM) [22]. Some of the grading scales usually used in clinical practice to evaluate peripheral neuropathy associated with chemotherapy include Total Neuropathy Score clinical version (TNSc), National Cancer Institute-Common Toxicity Criteria (NCI-CTC), modified Inflammatory Neuropathy Cause and Treatment (INCAT) group sensory sum score (mISS), European Organization for Research and Treatment of Cancer (EORTC) QLQ-C30, and CIPN (chemotherapy-induced peripheral neuropathy) 20 quality-of-life measures [23–27] (Table 3).

**Table 3:** Tools used for assessing OIPN

OIPN	Tools	References
Current tools used for the clinical diagnosis of OIPN	National Cancer Institute-Common Toxicity Criteria (NCI-CTC)	[23]
	Total Neuropathy Score clinical version (TNSc)	[24]
	Modified Inflammatory Neuropathy Cause and Treatment (INCAT) group sensory sum score (mISS)	[25]
	European Organization for Research and Treatment of Cancer (EORTC) QLQ-C30	[27]
	CIPN (chemotherapy induced peripheral neuropathy) 20 quality-of-life measures	[26]
	Skin biopsy and quantified intraepidermal nerve fiber density	[28, 29]
	LDI technology	[22]
	Surface electromyography recording	[30]
Comments for the ideal CIPN assessment tools	Specific to CIPN; validated in different types of chemotherapy; easy for patients to understand and complete; can be used in postal questionnaires as well as face to face; performs consistently in different settings; requires minimal training in use and scoring; sensitive to change; detects CIPN early; and reliable with minimal interrater variability	[2]

*CIPN* Chemotherapy-induced peripheral neuropathy, *LDI* Laser Doppler imager, *OIPN* Oxaliplatin-induced peripheral neuropathy

Furthermore, rapid non-invasive corneal confocal microscopy, LDI (laser Doppler imager) technology, and surface electromyography recording are also methods that can be used to confirm the diagnosis of CIPN in patients [22, 28–30] (Table 3). The combination of artificial intelligence (AI) with the traditional scales may be important in the management and understanding of the epidemiology of OIPN [31, 32]. While clinical examination is an important part of the assessment, it may pose some challenges in non-specialist settings, particularly where a more detailed sensory profiling is used for a definitive diagnosis [33, 34].

### Risk factors and genetic polymorphisms with OIPN

OIPN has become an increasingly significant clinical issue among cancer survivors [35]. Identifying who is at higher risk of developing OIPN would thus be an important step forward. Although there may be some statistical bias introduced by the techniques used, studies using multivariate statistical modeling have identified several risk factors, including age, medication (cardiovascular medication especially beta-blockers and the use of opioids), comorbid health conditions, raised body mass index (BMI), smoking, low serum albumin, neurofilament light chains in plasma or serum samples, decreased creatinine clearance, and baseline neuropathy [2, 36, 37] (Table 4, Fig. 2). It also may be possible to use detailed phenotyping (QST identified subclinical deficits, thermal hyperalgesia, and individuals more sensitive to chemotherapy) to identify preexisting vulnerabilities for developing OIPN [38–40].

**Table 4:** Risk factors and genetic polymorphisms associated with OIPN

OIPN	Comments	Reference
Risk factors associated with OIPN	Age; comorbid health conditions: decreased creatinine clearance, smoking, etc.; raised BMI; low serum albumin; and baseline neuropathy	[2, 4, 41]
	Medication: cardiovascular especially beta blockers; use of opioids	[42, 43]
	Quantitative sensory testing deficits in patients	[38, 39]
	Thermal hyperalgesia	[40]
	Neurofilament light chains in plasma or serum samples	[36, 37]
Genetic polymorphisms associated with OIPN	GSTP1 gene (Ile105Val polymorphism), cytochrome P450 enzymes, OCT2, ABCC2, and AGXT	[44–50].
	SCN4A (rs2302237); SCN10A (rs1263292) associated with an increased incidence of acute oxaliplatin-induced CIPN	[51]
	SCN9A (rs6746030) protected against severe oxaliplatin-induced CIPN	[52]

*ABCC2* ATP binding cassette subfamily C member 2, *AGXT* Alanine glyoxylate aminotransferase, *BMI* Body mass index, *CIPN* Chemotherapy-induced peripheral neuropathy, *GSTP1* Glutathione S-transferase pi 1, *OCT2* Organic cation transporter 2, *OIPN* Oxaliplatin-induced peripheral neuropathy, *SCN10A* Sodium channel protein type 10 subunit alpha, *SCN4A* Sodium channel protein type 4 subunit alpha, *SCN9A* Sodium channel protein type 9 subunit alpha

Genetic risk factors may be relevant for OIPN development, both in clinical settings and rodent models [53, 54]. It was suggested that polymorphisms of glutathione transferases (Ile105Val polymorphism of the GSTP1 gene), ATP binding cassette transporters, OCT2, cytochrome P450 enzymes, polymorphism of ABCC2 (ATP binding cassette subfamily C member 2), and AGXT (alanine glyoxylate aminotransferase) may be involved in the development of OIPN [44–50]. The development of acute OIPN was altered in patients with single-nucleotide polymorphisms in the SCNA genes encoding selected Na_v_ channels (Table 4, Fig. 2) [51, 52]. As mentioned, these genetic risk factors can influence the absorption and metabolism of OHP, which are associated with OIPN formation. Moreover, genotypic profiles of CIPN patients and the knowledge of genetic susceptibility needs to be incorporated into clinical trials.

## The molecular basis

**Fig. 3:**
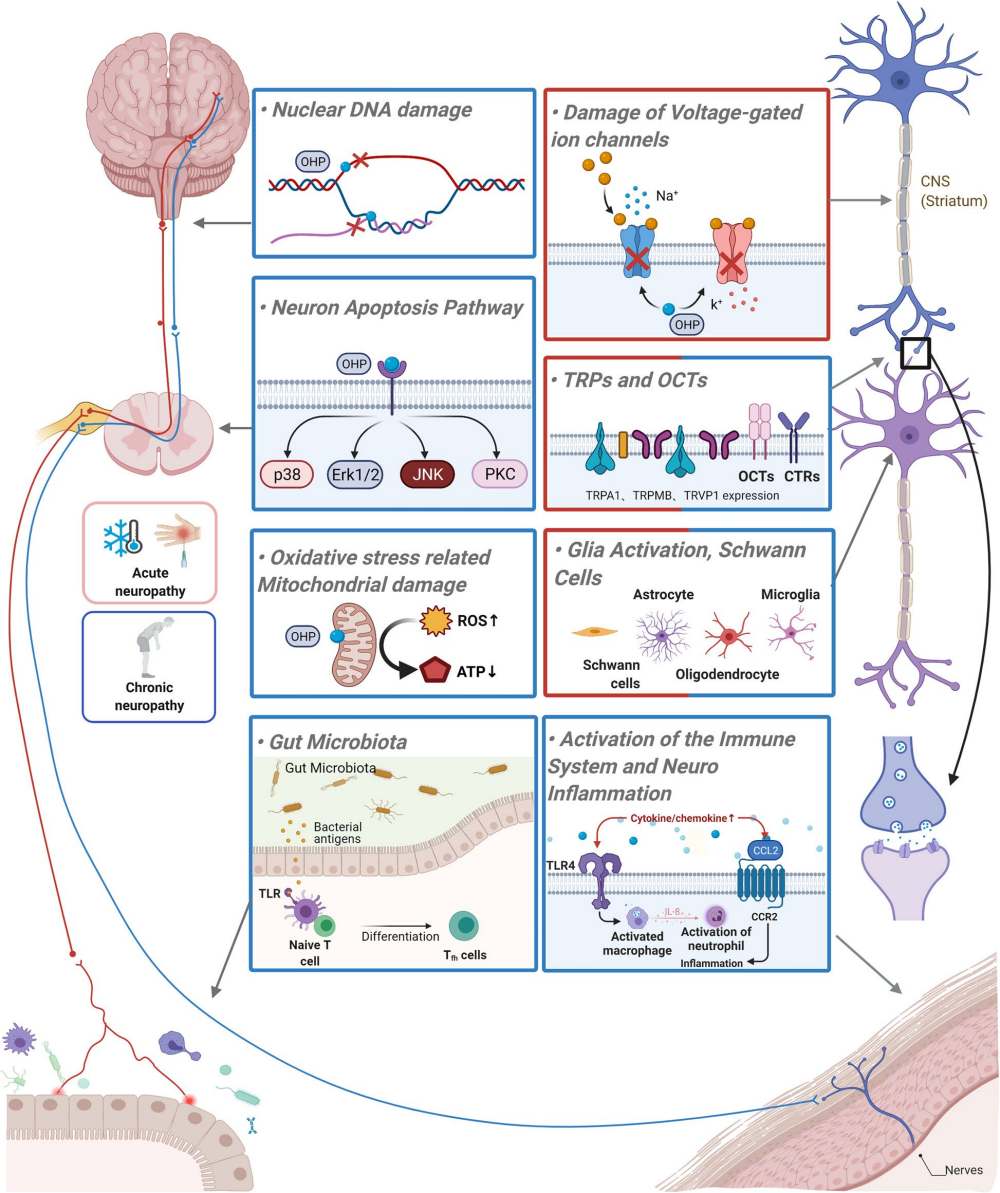
Mechanism schematic diagram of OHP-induced acute and chronic neuropathy. The red labeled box shows mechanisms that are the main reason of acute OHP-induced neuropathy. The blue labeled box shows mechanisms that are the main reason for chronic OHP-induced neuropathy. The mixed color box shows mechanisms involved in both acute and chronic OHP-induced neuropathy

The clinical features of OIPN provide important clues in the understanding of the basic molecular mechanisms of its onset. It is noteworthy that the mechanisms of acute and chronic OIPN are not identical. For acute OIPN, the dysfunction of ion channels, OCT protein, and glial cells are involved in the acute pain, whereas the main mechanisms relevant to chronic OIPN are nuclear DNA damage, oxidative stress-induced mitochondrial damage, glia activation-related neuroinflammation, and gut microbiota-induced inflammation (Fig. 3, Table 5). Mechanisms relying on the unique structure and function of the neurons need emphasis. The treatment of neuropathy should be modified with the discovery of new mechanisms.

**Table 5:** A summary of the possible mechanisms involved in the development of oxaliplatin-induced neuropathic pain

Targets	Mechanisms	References
Na^+^ channel	Prolonged open state and slow inactivation of the Na^+^ channels in acute OIPN	[12, 55–57]
	Induced abnormalities of Na^+^ currents in chronic OIPN	[58]
K^+^ channel	Increasing the expression of the pro-excitatory K+ channels	[59]
	Decreased expression of two-pore domain K^+^ channels (TREK-1 and TRAAK) in DRG	[60, 61]
	CAG repeat polymorphisms in the KCNN3 gene	[62]
Ca^2+^ channel	Oxalate as a calcium chelator contributes to the acute form of OIPN	[63]
	Increased expression of the Cavα2δ – 1 subunit mRNA and protein in cold hypersensitivity	[64, 65]
	Reduction in P/Q-, T-, and L-type Ca_v_ channel currents	[66]
Transient receptor potential channels	Up-regulation of the mRNA of the TRPV1, TRPA1, and TRPM8 in cultured DRG neurons	[67]
	OHP-induced cold allodynia in vivo was found to enhance the sensitivity and expression of TRPM8 and TRPA1	[68, 69]
	Oxaliplatin and oxalate cause TRPA1 sensitization to ROS	[70, 71]
Transporters	CTRs (CTR1) and OCTs (OCT2) mediate the uptake of OHP	[48, 72]
	ATP7A and ATP7B facilitate the cellular efflux of OHP	[73]
Nuclear DNA damage	Formation of platinum DNA adducts	[57, 74].
Oxidative stress-related mitochondrial damage	Neuronal mitochondrial dysfunction resulting in nitro-oxidative stress	[2, 75]
	Bind to mitochondrial DNA and formation of adducts	[76]
	Oxidative stress could gate TRPA1, produce nociceptive responses and neurogenic inflammation, and cause demyelination and disruption of the cytoskeleton of peripheral nerves	[77, 78]
	Lead to electron transport chain disruption and cellular energy failure in DRG neurons	[79]
	Nrf2 may play a critical role in ameliorating OIPN	[67, 80]
Activation of the immune system and neuroinflammation	Increased levels of CCL2 and CCR2 accompanied by mechanical hypersensitivity	[81]
	IL-8 signaling pathway is involved in neuroinflammation	[82]
	Gut microbiota -TLR4 activation on macrophages	[83]
	Increased circulating CD4 ^+^ and CD8 ^+^ T-cells	[84]
Glia activation	Increase of neuro-immune activation resulting in converted neurotransmission	[85–89]
	Transient activation of microglia and astrocytes in the spinal cord and supraspinal areas	
Schwann cells	Mitochondrial dysfunction in Schwann cells	[90]
Central nervous system structures and neurotransmitters	Altered levels of neurotransmitters, such as catecholamines, histamine, serotonin, glutamate, and GABA	[91–93]
	GLT-1 and GLAST and EAAT1 dysfunction	[94, 95]
Caspases and MAP-kinases, Protein kinase C, and PI3K/Akt2 pathway	Early activation of the MAP-kinase proteins p38 and ERK1/2, which promotes apoptosis-mediated cell death in rat DRG neurons	[96]
	Up-regulates the gamma isoforms of PKC and increases in the phosphorylation of gamma/epsilon PKC isoforms	[97]
	PI3K/Akt2 activation	[98]
MicroRNA regulation	MiR-15b down-regulation of BACE1 contributes to chronic neuropathic pain	[99]
Gut microbiota	Different microbe-associated molecular patterns (MAMPs) bind to their TLRs	[100]
	LPS can directly mediate the gating of TRPA1 and increase calcium influx	[101, 102]
	Chemotherapy decreased numbers of “beneficial” bacteria, such as *Lactobacillus* and *Bifidobacteria*, while *Lactobacillus acidophilus* exerts anti-tumor effects while preventing the incidence of the toxic adverse events	[103, 104]
	Microbiome-gut–brain and the neuroimmune–endocrine axis involved in the manifestations of OIPN	[103]

*ATP7A* ATPase Copper Transporting Alpha, *BACE1* Beta-secretase 1, *Cavα2δ – 1* Calcium voltage-gated calcium channel alpha2/delta subunit, *CCL2* C-C motif chemokine 2, *CCR2* C-C-Motif Receptor 2, *CD4 +* Cluster of Differentiation 4 receptors, *CD8 +* Cluster of Differentiation 8 receptors, *CTRs* Copper transporters, *DNA* Deoxyribonucleic acid, *DRG* Dorsal root ganglion, *DRG* Dorsal root ganglion, *ERK1/2* Extracellular regulated kinase 1/2, *GABA* γ-aminobutyric acid, *GLAST* EAAT1, glutamate aspartate transporter, *GLT-1* Glutamate transporter 1, *IL-8* Interleukin-8, *KCNN3* Potassium channel SK3, *LPS* Lipopolysaccharides, *MAMPs* Microbe-associated molecular patterns, *OCTs* Organic cation transporters, *OIPN* Oxaliplatin induced peripheral neuropathy, *PI3K/Akt2* Phosphatidylinositol 3 kinase/ protein kinase B, *PKC* Protein kinase C, *ROS* Reactive oxygen species, *TLR* Toll-like receptors, *TLR4* Toll-like receptors 4, *TRAAK* TWIK-related arachidonic acid-stimulated K+ channel, *TREK-1* TWIK—Related K+ channel 1, *TRPA1* Transient receptor potential A1, *TRPM8* Transient receptor potential cation channel subfamily M member 8, *TRPV1* Transient receptor potential vanilloid 1

### Ion channels dysfunction

#### Na^+^ channel

The Na^+^ channels play a core role in OHP-induced cold hyperalgesia [55]. Moreover, acute OHP-induced aggravation of cold hypersensitivity can be relieved by the administration of Na^+^ blockers (lidocaine, mexiletine, carbamazepine, etc.) [56, 105]. OHP can delay Na^+^ channels inactivation, which may be enhanced by exposure to cold [56]. Accumulation of OHP metabolite oxalate severed as a calcium chelator is thought to cause acute neurotoxicity. It can affect the functional properties of the Na^+^ channels resulting in a prolonged open state of the Na^+^ channels and hyperexcitability of the DRG sensory neurons [12, 57]. Furthermore, 78% of chronic OIPN patients showed abnormalities in Na^+^ channels in clinical studies of chronic OIPN [45].

#### K^+^ channel

The K^+^ channels are involved in regulating sensory neuronal pain and excitability [106]. It was demonstrated that OHP-induced neuropathy can induce functional abnormalities by increasing the pro-excitatory K^+^ channels expression, such as the hyperpolarization-activated channels (HCNs) [59]. Furthermore, it was observed that OHP decreased the expression of the two-pore domain K^+^ channels (TWIK-Related K^+^ Channel 1 (TREK-1) and TWIK-related arachidonic acid-stimulated K^+^ channel (TRAAK)) in the DRG of rodents [107]. The activator of TREK-1 and TRAAK (riluzole) prevents OHP-induced motor and sensory deficits and attenuates the OPH-induced depression-like symptoms [60, 61]. While a slight association between OIPN and CAG repeat polymorphisms in the potassium channel SK3 (KCNN3 gene) gene was shown in a preclinical model, this was not demonstrated clinically [62]. Moreover, activation of slow axonal K^+^-channels (K_v_7) reduced the OHP-induced hyperexcitability [108].

#### Ca^2+^ channel

Calcium chelator-oxalate contributes to the development of an acute form of OIPN. The administration of Ca^2+^ gluconate and Mg^2+^ sulfate was shown to reduce subsequent OHP-induced neuropathy [63]. The crucial role of the calcium voltage-gated calcium channel alpha2/delta (Ca_v_α_2_δ − 1) subunit in the development of OHP-induced acute and delayed cold hypersensitivity has also been shown in rodents [64, 65]. Ca_v_ channel currents are reduced by OHP in a concentration-dependent manner. Acute OHP treatment leads to a reduction in the L-, T-, and P/Q-type Ca_v_ channel currents. In contrast, prolonged OHP exposure to DRG neurons significantly increases T- and L-type Ca_v_ channel currents. Increased T- and L-type Ca_v_ channel protein levels in DRG neurons have also been noted after OHP exposure [66]. Furthermore, treatment with Ca^2+^ channel blockers to limit the Ca^2+^ influx is employed clinically [63].

#### Transient receptor potential channels

OHP-induced cold allodynia was found to enhance the sensitivity and expression of transient receptor potential cation channel subfamily M member 8 (TRPM8) and transient receptor potential vanilloid1 (TRPA1) in vivo [67–69]. OHP and oxalate have been shown to cause reactive oxygen species (ROS)-related TRPA1 sensitization by inhibiting prolyl hydroxylases. OHP-induced TRPA1 sensitization to ROS is believed to be caused by enzyme inhibition, enabling ROS signals to convert into cold sensitivity by TRPA1 [70, 71]. Moreover, OHP induces the hydrogen peroxide rapid generation and evokes cysteine oxidation-dependent human TRPA1 (hTRPA1) activation mediated by ROS, and glutathione can prevent the observed calcium influx [66, 70, 109]. It has been demonstrated that TRPM8 blocking by the administration of capsazepine inhibits OHP-induced cold allodynia in mice [68]. However, the use of the selective TRPV1 blocker suggests that TRPV1’s antagonistic and partial agonistic actions do not affect OHP-induced cold allodynia [110].

### Transporters

Organic cation transporters (OCTs) and copper transporters (CTRs) can mediate the uptake of OHP through their influence on the OHP influx and efflux of DRG neurons [48, 72]. The OCT2 protein was detected in human and mouse DRGs [111]. Single-dose OHP increased the sensitivity to cold and mechanical stimulation significantly in WT mice, compared with OCT2 knockout mice, and it was required at the acute OHP-induced peripheral neuropathy onset. Over-expressions of copper transport 1 (CTR1) and OCT2 in DRG neurons have resulted in OHP accumulation, leading to aggravation or development of the neuropathy [48]. ATPase Copper Transporting Alpha (ATP7A) can facilitate the OHP cellular efflux, reducing the possibility of OHP-DNA adducts forming. Thus, it was hypothesized that ATP7A-expressing DRG neurons are less sensitive to OHP-induced neuropathy. In contrast, high levels of CTR1 expressing DRG neurons would be expected to absorb more OHP, which could lead to toxic neuropathy effects [112].

### Nuclear DNA damage

Platinum is accumulated easily in the DRG neurons due to the abundant fenestrated capillary network and the lack of the blood–nerve barrier in the DRG [74, 113]. The accumulation of platinum–DNA adducts formation is considered the key factor in OIPN development [57, 74]. A correlation between platinum–DNA adduct levels and the degree of neuropathy has been identified [96]. Cisplatin-produced adducts were approximately three times higher than those generated by equimolar OHP doses, which in accordance with cisplatin caused significantly greater neuronal cell deaths than OHP in vitro [96]. The results may explain the symptom that OHP-induced neuropathy showed improved outcomes compared with other platinums.

### Oxidative stress-related mitochondrial damage

Neuronal mitochondrial dysfunction resulting in nitro-oxidative stress [114] plays a critical role in OHP-induced neuropathy [2, 75]. Due to the lack of DNA repair systems, OHP-DNA adducts cannot be repaired within the mitochondria. Evidence has shown the relationship between oxidative stress and OHP-induced neuropathy [76]. Furthermore, OHP causes damage to both neuronal and nonneuronal mitochondria, leading to oxidative stress burden mediated by the redox-sensitive TRPA1 channels, which correlate with OHP-induced cold and mechanical hypersensitivity [71, 115–117]. The ROS generated by OHP treatment has been shown to modulate sodium channel activity, influencing the sensitivity of nociceptors [118]. Alterations in the mitochondrial structure and function in OHP-exposed rat neuronal cells have been shown in vitro [119, 120]. Moreover, OHP can produce toxic effects on axonal mitochondria, which lead to electron transport chain dysfunction and failure of cellular energy in the DRG neurons [79]. The antioxidant Acetyl-l-Carnitine treatment inhibits the OHP- triggered hyperalgesia development by preventing the respiratory chain damage, further leading to the maintenance of mitochondrial integrity [79]. However, the effects of antioxidants (vitamin C, etc.) in OIPN require further clinic tests and verifications.

### Activation of the immune system and neuroinflammation

The mRNA levels of proinflammatory cytokines and chemokines increase accompanied by OHP administration, and a strong correlation of this effect with the mechanical hypersensitivity development has been shown [81]. The IL-8 signaling pathway is involved in neuroinflammation, resulting in progressive neural sensitization in OIPN model [82]. Increased levels of C-C motif chemokine 2 (CCL2) and C-C-Motif Receptor 2 (CCR2) in the DRG neurons have also been observed to be accompanied by a mechanical hypersensitivity in OHP-treated rats [121]. Toll-like receptors (TLR) are widely expressed on immune cells, enterocytes, sensory neurons, and glial cells. OHP-induced mechanical hyperalgesia and neuroinflammation are mediated by gut microbiota-related Toll-like receptors 4 (TLR4) activation on macrophages [83, 122, 123]. T-cells (Th17 and Th1) are also involved in the possible sources of inflammatory factors and potential drivers of neuropathic pain. In OHP-treated male mice, significant mechanical allodynia was accompanied by an increased circulating cluster of differentiation 4 receptors (CD4^+^) and cluster of differentiation 8 receptors (CD8^+^) T-cells [84]. Since chemotherapy-associated inflammation is regarded as a key mechanism of neuroinflammation involved in OIPN [84], the complex interaction between neurons, immune system, and cancer cells must be considered.

### Glia activation and Schwann cells

Abnormal communication between the neurons and the glia plays a significant role in OHP-induced neuropathic pain [124]. This dysfunction can arise from the increasing neuro-immune activations, resulting in converted neurotransmissions within the dorsal horn of the spinal cord. This has been substantiated by the evidence that pharmacological treatments (i.e., minocycline [85] and fluorocitrate [86]) can relieve neuropathic pain through the prevention of glial activation. It has been shown that following the intraperitoneal administration of OHP, transient activation of the microglia and the astrocytes in supraspinal areas and the spinal cord is involved in the modulation of pain accompanied by a decrease in the thermal and mechanical pain thresholds [87]. During neuropathy, the numbers of ionized calcium binding adapter molecule 1 (Iba1) (microglia) and glial fibrillary acidic protein (GFAP) (astrocyte) immune-positive cells showed an increase in the dorsal horn of the spinal cord, concomitantly with a decrease of pain threshold and a glia density increase in various supraspinal sites [88]. The uptake and metabolism process of OHP in glia cells and neurons should be considered. Furthermore, OHP induces intra-epidermal nerve fibers loss and moderate axonal degeneration in patients [125]. The OHP-induced cytotoxicity in primary Schwann cells and the myelin basic protein expression decreasing indicated that the platinum derivatives also induced Schwann cells mitochondrial dysfunction in vitro [90].

### Central nervous system structures and neurotransmitters

Altered levels of several neurotransmitters are associated with OIPN [91–93]. In OHP-treated rats, the blockade of proinflammatory cytokine receptors results in γ-aminobutyric acid (GABA) function recovery and the cold and mechanical hypersensitivity relief [126]. These transport proteins have been observed in OHP-treated neuropathic rats [94]. Single-dose and repeated doses of ceftriaxone, a beta-lactam antibiotic upregulating glutamate transporter 1 (GLT-1) expression, increases glutamate reuptake in the central nervous system (CNS) [127, 128]. It was tested for effectiveness in relieving early-phase and late-phase mechanical and thermal hyperalgesia in OHP-treated mice. The results confirmed previous findings, which suggested that the GLT-1 biological functions are associated to a greater extent with the regulation of the mechanical- than the cold-nociceptive threshold [129]. The effect of tiagabine, a highly selective inhibitor of GABA transporter subtype 1 (GAT-1), on OHP-induced neuropathic pain was assessed in mice [130]. However, the central effects of chemotherapy neurotoxicity are mainly related to cognitive impairment (“chemofog” or “chemobrain”) of the central nervous system in OIPN [88, 131, 132]; it is important to investigate the central nervous system with magnetic resonance imaging at the functional level or to modulate its activity using transcranial stimulation to develop new therapeutic strategies [133].

### Neuron apoptosis pathway

Prolonged OHP exposure induces the phosphoprotein 38 (p38) and extracellular regulated kinase 1/2 (ERK1/2) early activation, which promotes the apoptosis of DRG neurons, and the down-regulation of protective c-JunNH2-terminal kinase /stress activated protein kinase (JNK/Sapk), which increase OHP neurotoxic effects in vitro [96, 134]. This was further evidenced by using a caspase inhibitor (z-VAD-fmk), which indicated the involvement of caspases in OHP-induced neuropathy. Furthermore, restoring the MAP-kinases’ physiological functions, through the treatment of the DRG neurons with retinoic acid or NGF, is neuroprotective against OHP-induced neuropathy in vitro [135]. The gamma isoforms of protein kinase C (PKC) and the phosphorylation of gamma/epsilon PKC isoforms are increased in the brain in an OIPN model, and PKC inhibitors (Calphostin C, hypericin) attenuate OHP-induced mechanical hyperalgesia [97]. A cyclooxygenase-2 (COX-2) inhibitor (celecoxib) inhibits the phosphatidylinositol 3 kinase/ protein kinase B (PI3K/Akt2) pathway and can also attenuate OHP-induced neuropathic pain [98].

### Gut microbiota

Recent studies have demonstrated that gut microbiota is involved in pain modulation [136], and the different types of peripheral neuropathies induced by OHP in both the c mice and germ-free (GF) mice have indicated that the gut microbiota is critical to the induction and pathogenesis of OIPN [83]. Mechanically different microbe-associated molecular patterns (MAMPs) binding to the TLRs activate resident immune cells with the release of numerous chemokines and cytokines by the immune cells, which subsequently alters the transmission and transduction of nociceptive sensory neurons [100]. Moreover, lipopolysaccharides (LPS) can mediate the gating of TRPA1 directly and increase calcium influx [101, 102]. The gut microbiota serves as a connection between the microbiome-gut–brain and the neuroimmune–endocrine axes, forming a complex network that can influence main components involved in the symptoms of OIPN directly or indirectly [103]. Patients always receive antibiotic prophylaxis to prevent infection before chemotherapy onset in clinical settings. However, the necessity of this antibiotic prevention and the type of patient suitable for this treatment are not clear. The antibiotics may imbalance the gut microbiota and aggravate the OIPN. The instructions for antibiotic prophylaxis need to be evaluated in order to prevent the unexpected side effects of chemotherapy. This also highlights the need for targeted therapeutically specific microflora for successful treatment regimens to be explored.

## Chemotherapy-induced peripheral neuropathy prevention and treatment: therapy candidates for OIPN

The National Cancer Institute’s Symptom Management and Health-Related Quality of Life Steering Committee has announced that CIPN is a priority area in translational research in cancer care [137]. However, based on the background described previously, this is not yet completely feasible. Current pharmacological approaches do not have a sound rationale, and there is a strong medical demand for novel therapeutic regimens. Moreover, the latest literature data indicate clearly that although numerous preventive therapies have been tested for their potential utility to alleviate CIPN, it is currently still not preventable [138–140], and many strategies that were tested were found to be ineffective. Duloxetine is the only drug moderately recommended by the American Society of Clinical Oncology (ASCO) for the prevention of OIPN [1, 2]. Other drugs such as venlafaxine, pregabalin, and carbamazepine have also been assessed, but their utility for the prevention of oxaliplatin-induced CIPN is still controversial (Table 6). These guidelines show that due to the lack of high-quality and strong evidence for the action of the agents tested, there are no clear algorithms for CIPN prevention or treatment (Table 7).

**Table 6:** A summary of ASCO recommendations for preventative and treatment therapies for CIPN

Strength of recommendation	Preventative therapies	Treatment therapies
Strong recommendation against	Acetyl-L-carnitine	None
Moderate recommendation against	Acetylcysteine, Amifostine, Amitriptyline, Calcium and magnesium, Cannabinoids, Calmangafodipir, Carbamazepine/oxcarbazepine, L-carnosine, DDTC, Gabapentin/pregabalin, Glutamate/glutamine, GSH, GJG–Kampo medicine, Metformin, Minocycline, Nimodipine, Omega 3, Org 2766, Retinoic acid, rhuLIF, Venlafaxine, Vitamin B, Vitamin E	None
Inconclusive date: No recommendation	Acupuncture, Compression therapy, Cryotherapy, Exercise, GM1,	Acupuncture, Exercise, Gabapentin/pregabalin, BAK, Oral cannabinoids, Tricyclic antidepressants, Scrambler therapy
Moderate recommendation for	None	Duloxetine
Strong recommendation for	None	None

*BAK* Topical amitriptyline, ketamine, 6 baclofen, *CIPN* Chemotherapy-induced peripheral neuropathy, *DDTC* Diethyldithiocarbamate, *GJG* Goshajinkigan, *GM1* Monosialotetrahexosylganglioside, *GSH* Glutathione, *rhuLIF* Recombinant human leukemia inhibitory factor

**Table 7:** Emerging drug candidates tested in clinical trials for the prevention and treatment of OIPN

Agent	Mechanism of action/targets	Clinical trial number/ PubMed Unique Identifier: Status/Findings
*Ion channel-targeted therapies*		
Riluzole	Prevents the excessive accumulation of glutamate [94] Interaction with potassium channels of the K2P family (TREK, TRAAK) [141]	NCT03722680: Recruiting NCT04761614: Not yet recruiting
Lidocaine	Blocks sodium channels [142]	NCT03254394: Active, not recruiting PMID 28458593: Intravenous lidocaine has a direct analgesic effect in CIPN with a moderate long-term effect and seems to influence the area of cold and pinprick perception. Additional research is needed, using a control group and larger sample sizes to confirm these results [143].
Pregabalin	Blockade of voltage-gated calcium channels [144]	NCT01450163: Completed:The preemptive use of pregabalin during OHP infusions was safe, but did not decrease the incidence of chronic pain related to OIPN. NCT02394951: Completed
Calcium and Magnesium Infusion	Intravenous delivery of calcium and magnesium facilitates the action of sodium channels, thereby blocking them [145]	PMID 21067912: Ca/Mg infusions significantly reduced all grade oxaliplatin-related neurotoxicity [146]. PMID 21189381: Intravenous Ca/Mg as an effective neuroprotectant against oxaliplatin-induced cumulative sNT in adjuvant colon cancer [147]. NCT01099449: This study does not support using calcium/magnesium to protect against oxaliplatin-induced neurotoxicity (Completed) [148]. PMID 24156389: Ca/Mg infusions do not alter the clinical pharmacokinetics of oxaliplatin and do not seem to reduce its acute neurotoxicity [149].
*Neurotransmitter-based therapy*		
Duloxetine	Serotonin-noradrenaline reuptake inhibitor [150]	NCT04137107: Recruiting NCT03812523: Not yet recruiting NCT00489411: Duloxetine-treated patients with high emotional functioning are more likely to experience pain reduction (*p* = 0.026) (Completed). NCT00489411: Among patients with painful chemotherapy-induced peripheral neuropathy, the use of duloxetine compared with a placebo for 5 weeks resulted in a greater reduction in pain (Completed). PMID 30105459: Duloxetine seems to be more effective than venlafaxine in decreasing the symptoms of chemotherapy-induced peripheral neuropathy. Duloxetine was more effective than venlafaxine in decreasing motor neuropathy and neuropathic pain grade [141].
Venlafaxine	Serotonin-noradrenaline reuptake inhibitor [144, 151]	NCT01611155: The present study neither supports the use of venlafaxine for preventing oxaliplatin-induced neuropathy in clinical practice nor the initiation of a phase III trial to investigate venlafaxine in this setting (Completed). PMID 21427067: Venlafaxine has clinical activity against oxaliplatin-induced acute neurosensory toxicity [152].
*Antioxidants*		
Amifostine	Prodrug that is dephosphorylated by alkaline phosphatase in tissues to a pharmacologically active free thiol metabolite [55]	NCT00601198: Terminated PMID 12960114: Amifostine, at a dose of 1000 mg, is better tolerated when administered s.c. Switching to the s.c. route in patients with poor tolerance and using i.v. administration allows the continuation of cytoprotection with minor side effects. Although preliminary, 1000 mg of amifostine effectively protected against the lower, still more frequently administered doses of chemotherapy given once every 2 weeks [153, 154].
Calmangafodipir	Mitochondrial MnSOD mimetic that reduces ROS tissue levels [155, 156]	NCT00727922: Mangafodipir can prevent and/or relieve oxaliplatin-induced neuropathy in cancer patients (Completed). NCT04034355: Completed NCT03654729: Completed NCT01619423: Calmangafodipir at a dose of 5 μmol/kg appears to prevent the development of oxaliplatin-induced acute and delayed CIPN without apparent influence on tumor outcomes (Completed).
*Anti-Inflammatory Therapy*		
Minocycline	A microglia inhibitor and a MMP9 blocker, inhibits the release of proinflammatory cytokines and alleviates the development and symptoms of OIPN [157, 158]	PMID 28551844: Results of this pilot study do not support the use of minocycline to prevent CIPN, but suggest that it may reduce P-APS and decrease fatigue; further study of the impact of this agent on those endpoints may be warranted [159].
*Targeting Chemotherapeutic Drug Uptake Transporters*		
Dasatinib	Targeting chemotherapeutic drug uptake transporters: OCTN2 inhibitor [150]	NCT04164069: Recruiting
*Targeting apurinic/apyrimidinic endonuclease function*		
APX3330 APX2009	Targeting apurinic/apyrimidinic endonuclease function: Enhance APE1 expressio n[160]	PMID 27608656: APX3330 and APX2009 might be effective in preventing or reversing platinum-induced CIPN without reducing the anticancer activity of platinum-based chemotherapeutics [160]. NCT03375086: Completed
*Targeting the Inhibition of Neuronal Apoptosis and Astrocyte Activation*		
Fingolimod	Targeting the inhibition of neuronal apoptosis and astrocyte activation: S1PR1 antagonism [161]	PMID 31882542: The development of a specific S1P2 agonist may represent a promising therapeutic approach for the management of chemotherapy-induced neuropathy [162]. NCT03943498: Recruiting
*Sigma 1 Receptor Antagonism*		
MR309	Sigma 1 receptor antagonism [163]	PMID 28924870: A Randomized, Double-Blind, Placebo-Controlled Phase IIa Clinical Trial: Intermittent treatment with MR309 was associated with reduced acute OIPN and higher oxaliplatin exposure, and showed a potential neuroprotective role for chronic cumulative oxaipn. Furthermore, MR309 showed an acceptable safety profile [164].
*Angiotensin II Type 2 Receptor Antagonism*		
EMA401 (Olodanrigan)	Angiotensin II type 2 receptor antagonism [165]	EudraCT Number: 2011–004033-13
*Carbonic Anhydrase Inhibitor*		
Topiramate Acetazolamide	Carbonic anhydrase inhibitor	PMID 31634341: topiramate and acetazolamide; revert oxaliplatin-induced acute cold allodynia in mice while not affecting OHP-induced cytotoxicity in cancer cells [166].
*Lipid Peroxidation Inhibitors*		
L-Carnosine	Scavenge the reactive oxygen species (ROS) formed by excessive oxidation of fatty acids and α-β unsaturated aldehydes [167]	PMID: 30592963: L-Carnosine exerted a neuroprotective effect against oxaliplatin-induced peripheral neuropathy in colorectal cancer patients by targeting Nrf-2 and NF-κB pathways [168].
GM1	Neuroprotective, neurotrophic-factor-like activity by activating the Trk neurotrophin receptors, prevent seizures and oxidative stress [169, 170]	NCT02251977: Patients receiving GM1 were less troubled by the symptoms of acute neuropathy. However, we do not support the use of GM1 to prevent cumulative neurotoxicity (Completed).
*5-HT2C Receptor Agonists*		
Lorcaserin	5-HT_2C_ receptor agonist [171]	NCT04205071: Withdrawn NCT03812523: Not yet recruiting
TRK-750		NCT04282590: Not yet recruiting
*Non-Pharmacological Studies*		
General management	Dose reductions in patients Delay the cycle of therapy	PMID 25417732: Cumulative dose of oxaliplatin is associated with long-term CIPN. The risk of developing long-term CIPN could only be reduced by decreasing the cumulative dose, whereas probable delay is not beneficial. Patients receiving a dose reduction because of acute neuropathy are still at risk of developing long-term CIPN. Future studies should focus on identifying patients who are at risk of developing CIPN [172].
rTMS	A noninvasive form of brain stimulation in which a changing magnetic field is used to provide electric current at a specific area of the brain through electromagnetic induction [173].	NCT03219502: Recruiting
Strength and Balance Training Program	Lifestyle-related factors can aid in preventing or reducing the neurological side effects of chemotherapy	NCT01422993: Completed
Diet	Polyamine-deprived diet	NCT01775449: Completed
Henna Application	Herbal extracts used in the treatment of diabetic cutaneous ulcers [174]	NCT04201587: Completed

*APE1* Apyrimidinic endonuclease/redox effector factor, *CIPN* Chemotherapy-induced peripheral neuropathy, *GM1* Monosialotetrahexosylganglioside, *MMP9* Matrix Metallo-peptidase 9, *MnSOD* Manganese superoxide dismutase, *OCTN2* Organic cation transporter-2, *OHP* Oxaliplatin, *OIPN* Oxaliplatin-induced peripheral neuropathy, *ROS* Reactive oxygen species, *rTMS* Repetitive Transcranial Magnetic Stimulation, *S1P2* Sphingosine-1-phosphate receptor2, *S1PR1* Sphingosine-1-Phosphate Receptor 1, *TRAAK* TWIK-related arachidonic acid-stimulated K^+^ channel, *TREK* TWIK-Related K^+^Channel

### Ion channel-targeted therapies

#### Riluzole

Riluzole prevents the excessive glutamate accumulation in OIPN, and benefits sensorimotor and painful disorders of the peripheral nervous system [94]. It has been shown to alleviate OHP-induced peripheral nerve dysfunctional and morphological alterations [61]. It also has been hypothesized that riluzole may exert its neuroprotective action through interaction with the potassium channels TREK and TRAAK [175]. Recently, riluzole has initiated a phase II, placebo-controlled, randomized, double-blind, parallel, multicenter, prevention trial with adult stage II/III colorectal cancer patients treated with a simplified FOLFOX4 regimen in the adjuvant setting (NCT03722680).

#### Lidocaine

Lidocaine is a sodium channel antagonist [142], and it was first assessed to revealed a significant alleviation of the cold and mechanical allodynia induced by OHP in rodent models [56, 176]. In a small study, the IV administration of lidocaine had a direct analgesic effect in CIPN and a moderate long-term effect through modulation of the cold and pinprick perception [143]. A pilot study was conducted to determine the tolerative and effective of IV lidocaine treatment to reduce the severity of OHP-induced cold hypersensitivity in modified FOLFOX6 (mFOLFOX6) chemotherapy patients. However, there remains a lack of convincing evidence supporting its efficacy [177].

#### Pregabalin

Pregabalin displays an anti-nociceptive effect by voltage-gated calcium channels blockade and the down-regulation of excitatory neurotransmitters [144]. A powerful analgesic effect of pregabalin on OIPN has been reported [178]. In three clinical cases, pregabalin successfully demonstrated a therapeutic effect; however, it was accompanied by similar side effects to those of gabapentin [179]. Oral administration of pregabalin reduced grades 1–2 severity of sensory neuropathy induced by OHP. However, in a Phase III trial (143 patients), the pre-administration of oral pregabalin during OHP infusion did not improve the chronic pain, life quality, or mood of the cancer patients [180]. Thus, the efficacy of pregabalin against CIPN requires further confirmation. In a current Phase III study, pregabalin was administrated exclusively for 3 days before and after the OHP infusion; it was able to prevent the occurrence of pain secondary to both the acute and chronic OIPN (NCT01450163).

#### Calcium and magnesium infusions

Calcium and magnesium (Ca/Mg) infusions are promising strategies for preventing OIPN. The intravenous delivery of calcium and magnesium facilitates the blocking of sodium channels [145]. In a large phase III study (720 advanced colorectal cancer patients/551 patients received Ca/Mg infusions before chemotherapy), Ca/Mg infusion decreased all grade incidence of the sensory neurotoxicity induced by OHP [146, 147]. However, in a double-blind phase III study (involving 353 colon cancer patients), intravenous Ca/Mg showed no benefit regarding the incidence of OHP-induced acute neurotoxicity symptoms when compared with a placebo [148]. A further two cases showed that Ca/Mg infusions altered neither the acute nor the chronic neurotoxicity induced by OHP [149]. Thus, the utility of Ca/Mg infusions should be examined further.

### Neurotransmitter-based therapies

#### Duloxetine

The antidepressant drug duloxetine, which acts as a serotonin-noradrenaline reuptake inhibitor (SNRI), can effectively alleviate symptoms of OIPN without reducing the antitumor activity of OHP based on the preclinical and clinical studies [137, 150, 181]. While ASCO recommends the use of duloxetine as a potential treatment for CIPN, sufficient evidence is still lacking. Thus, identifying predictors of duloxetine response and optimizing the treatment schedule is a priority as it is not completely effective and works for everyone. Furthermore, the patients with OIPN are more likely to benefit from duloxetine than patients with a paclitaxel-induced neuropathy based on an exploratory responder analysis [182], which suggests that action of duloxetine’s pharmacodynamic effect may be tightly associated with specific molecular mechanisms underlying OIPN [183, 184]. In addition, the administration of duloxetine was reported to have fewer adverse effects compared with venlafaxine [184]. Moreover, duloxetine had a better effect on reducing neuropathic pain severity and grade of motor neuropathy than venlafaxine [141]. Recently, duloxetine has initiated a phase II/III study investigating the best dose of duloxetine and how well it worked in preventing pain symptoms caused by OHP in stage II–III colorectal cancer patients (NCT04137107).

#### Venlafaxine

Venlafaxine, a more selective SNRI, has been used to prevent CIPN [144]. In a randomized phase III trial, venlafaxine displayed clinical activity against acute neurosensory toxicity induced by OHP; however, its side effects should not be ignored, including asthenia (39.2%) and nausea (43.1%) [152]. A greater analgesia benefit of SNRIs was observed in platinum-treated than in taxanes-treated patients; however, clinical data showed that duloxetine may improve CIPN symptoms more than venlafaxine [185]. Moreover, a direct comparison between duloxetine and venlafaxine is necessary. Unfortunately, a randomized pilot study on 50 patients failed to demonstrate the efficacy of venlafaxine in CIPN patients [151].

### Antioxidants

#### Amifostine

Amifostine was first used in the prevention of OIPN in a randomized trial. Neuropathy was reported on days when OIPN patients experienced sensitive peripheral neuropathy or cold-associated paresthesia. Amifostine administration showed significantly less neuropathy in the treatment group, whereas, toxicity of amifostine was not addressed. The intravenous administration of amifostine can cause significant adverse effects, including nausea, hypotension, and vomiting. The subcutaneous route of amifostine can significantly improve its tolerance [153, 154].

#### Calmangafodipir

Targeting manganese superoxide dismutase (MnSOD) has emerged as a promising strategy to prevent OIPN symptoms [186, 187]. A derivative of mangafodipir, calmangafodipir (Ca4Mn(DPDP)5, PledOx®), which is a cytoprotectant agent and a magnetic resonance imaging contrast agent [155, 156], is a mitochondrial MnSOD mimetic that can reduce ROS tissue levels. However, these compounds have been confirmed to have significant neuroprotective and preventive activity in OIPN preclinical and clinical data [186, 187]. Furthermore, the neuroprotection effect of calmangafodipir may also be possible with other chemotherapeutic agents [137, 188]. In a phase II study in colorectal cancer patients treated with OHP, calmangafodipir reduced cold allodynia and other sensory symptoms without reducing its antineoplastic efficacy [188]. Two international trials (POLAR A and POLAR M) have been initiated to evaluate the efficacy of calmangafodipir in prevention of OHP-induced neuropathy in colorectal cancer patients. Results are expected in the years 2020/2021 (POLAR A) or 2021–2023 (POLAR M) [137].

### Anti-inflammatory therapies

#### Minocycline

Minocycline, a microglia inhibitor and a matrix metallopeptidase 9 (MMP9) blocker, inhibits the release of proinflammatory cytokines and alleviates the development and symptoms of OIPN [157, 158]. It has been reported that minocycline treatment effectively prevented the mechanical sensitivities and the loss of IENFs in OIPN models [189]. In 2017, a pilot study reported that minocycline did not reduce the CIPN overall sensory neuropathy. However, compared with the placebo, it decreased the average pain score and fatigue in CIPN patients [159]. Therefore, minocycline may be a promising candidate for the prevention and treatment of CIPN. Large clinical trials and preclinical studies are needed to further evaluate its effect on CIPN.

### Targeting chemotherapeutic drug uptake transporters

Two tyrosine kinase inhibitors, nilotinib (an organic anion transporting polypeptide 1B2 (OATP1B2) inhibitor) and dasatinib (an OCTN2 inhibitor), may provide a potential neuroprotective strategy for OIPN, without impacting negatively on their systemic clearances or antitumor efficacies through in vitro and in vivo studies. In a phase II study, nilotinib and dasatinib are currently being studied in an ongoing phase IB trial as repurposed drugs [137].

### Targeting apurinic/apyrimidinic endonuclease function

Impaired DNA repair within the sensory nervous system is associated with CIPN formation. The apyrimidinic endonuclease/redox effector factor (APE1) is an important enzyme for DNA-damaged base removal, and decreased APE1 levels in sensory neurons increase neurotoxicity in OIPN models, targeting APE1 by the small molecules APX3330 and APX2009. This has been shown to provide neuroprotection against OIPN. APX2009 also shown a strong effect of tumor cell-killing. These data suggest that such compounds may be effective in preventing or reversing platinum-induced neuropathy without affecting the anticancer ability of the platinum-based chemotherapies [190]. Currently, clinical trials are being conducted with APX3330, regarding CIPN prevention as an antineoplastic agent.

### Targeting the inhibition of neuronal apoptosis and astrocyte activation

OHP induces the dysregulating sphingolipid metabolism, and leads to increased formation of sphingosine-1-phosphate (S1P) in the development of CIPN [162]. Sphingosine kinase inhibitors reversed CIPN symptoms by blocking the formation of S1P [191]. Fingolimod (FTY720) acts as a nonselective agonist of SIP receptors. Daily FTY720 injections have been shown to inhibit the development of hyperalgesia and mechanical allodynia induced by OHP [162]. Similar effects were also found with other S1P1 antagonists. S1P1 antagonists may act synergistically without reducing the anticancer activity of chemotherapeutic agents [192], making fingolimod and its analogs promising agents for the prevention of OIPN [193]. Moreover, fingolimod-induced transient cardiovascular adverse effects must be considered for CIPN prevention and treatment [137].

### Sigma-1 receptor antagonism

The sigma-1 receptors may constitute a novel drug target candidates for OIPN [163], and MR309, a selective sigma-1 receptor antagonist, has attenuated symptoms of OIPN [194]. A phase II clinical trial with MR309 has been completed in patients with colorectal cancer receiving OHP therapy [194]. Compared with the placebo, MR309 significantly reduced the threshold of cold pain and lowered the proportion of patients with severe chronic neuropathy. Continuous dosing during the full chemotherapy period and different dose regimens of MR309 administration require testing in further studies [194].

### Angiotensin II type 2 receptor antagonism

EMA401 (olodanrigan) is an antagonist of angiotensin II type 2 (AT2) receptor. Oral EMA401was tested for effectiveness in CIPN patients in clinical trials. Although EMA401 showed anti-neuropathic properties in painful diabetic neuropathy and postherpetic neuralgia patient populations, these trials were terminated or withdrawn due to the observed side effects of EMA401 [93, 94, 126, 127]. An open-label biomarker study of EMA401 was conducted in a phase II clinical trial of OIPN patients to prove the conceptual use of EMA401 in OIPN. However, because the statistical analyses were not specified as only one arm was reported, the trial results were inconclusive and difficult to interpret.

### Carbonic anhydrase inhibitor

Therapeutically relevant concentrations of OHP have demonstrated a decrease in the DRG pH value in mice by forming adducts with hemoglobin in in vivo and in vitro experiments. Moreover, the FDA-approved drugs, i.e., acetazolamide and topiramate, alleviate OHP-induced acute cold allodynia in mice without affecting OHP-induced tumor-killing effect in vivo. The results indicate a novel strategy for future OIPN clinical treatment [195].

### Lipid peroxidation inhibitors

#### L-carnosine

L-carnosine, in combination with alpha-lipoic acid, showed a significant reduction in neuropathic pain and good tolerability in an animal model of OIPN. The mechanism of L-carnosine revealed that its calcium-binding carnosine moiety exerts a persistent activity through a TRPA1 synergically stabilized binding through covalent binding to the lipoic acid residue of the channel [196]. The prophylactic effect of exogenous L-carnosine in the prevention of oxidative stress was evaluated in an open-label prevention study, in which the chemotherapy patients received 500 mg per day L-carnosine orally, and the neuropathy grading score of the National Cancer Institute-Common Toxicity Criteria for Adverse Events was applied to assesses the peripheral neuropathy [197].

#### GM1

Monosialotetrahexosylganglioside (GM1) is important in nerve development, differentiation, and repair after injuries [169]. It also acts to prevent oxidative stress and seizures [170]. GM1 was initially applied to treat Parkinson’s disease and vasculogenic or traumatic central nervous impairments treatment [169]. GM1 attenuated the symptoms of acute neuropathy in a clinical trial conducted in colon cancer patients treated with FOLFOX therapy; however, its use in preventing neurotoxicity of OHP is currently not recommended. The utility of this agent in this clinical condition requires additional placebo-controlled studies [198].

### 5-HT_2C_ receptor agonists

Lorcaserin, a selective 5-HT_2C_ receptor agonist, has effects on a range of physiological functions and behaviors. Lorcaserin also has potential clinically relevant effects in models of pain and seizure-like activities [199]. The first trial, a randomized phase II study, compared lorcaserin with duloxetine in the treatment of chronic OIPN (NCT03812523). The second study, a phase I open-label trial, investigated how well lorcaserin works in treating CIPN in stage I-IV gastrointestinal or breast cancer patients (NCT04205071).

### Non-pharmacological studies

Extending the time of an intravenous drip to potentially avoid plasma peaks, rather than direct damage to nerve tissue, is possible [200]. Several clinical trials have been used in dose reductions in patients with incipient neuropathy signs/symptoms (85 mg/m^2^ OHP); when the neurologic examination was normal in the adjuvant setting, the dose reduction was from 85 to 75 mg/m^2^, and 65 mg/ m^2^ was applied in the metastatic setting [172]. The use of repetitive transcranial magnetic stimulation (rTMS) in headache and pain, and in other neurological and psychiatric conditions, has been proposed [173]. In a study, OIPN patients were randomized to be treated as follows: rTMS or sham rTMS over 30 min for 10 sessions over 10 business days, or standard of care (NCT03219502). Furthermore, change of lifestyle-related factors can aid in reducing or preventing the OIPN neurological side effects. The clinical study evaluated the effects of an exercise program designed to improve lower extremity balance and strength in OIPN persons with a specific purpose (NCT01422993), on their balance, strength, and neuropathic symptoms. However, the current evidence is weak to support the hypothesis, and serious limitations have been found in many studies reporting positive results [160]. The Non-Pharmacological Study also aimed at determining whether a polyamine-deprived diet (a specific nutritional therapy) can prevent acute OIPN in patients receiving FOLFOX4 (NCT01775449). Among herbal extracts, henna is used to treat diabetic cutaneous ulcers with small nerve fiber loss [174]. Moreover, the administration of different anticancer drugs, including OHP, also showed this pathological event, and the preliminary effects of henna on CIPN have been evaluated (NCT04201587).

### Outlook of future drug candidates

**Fig. 4:**
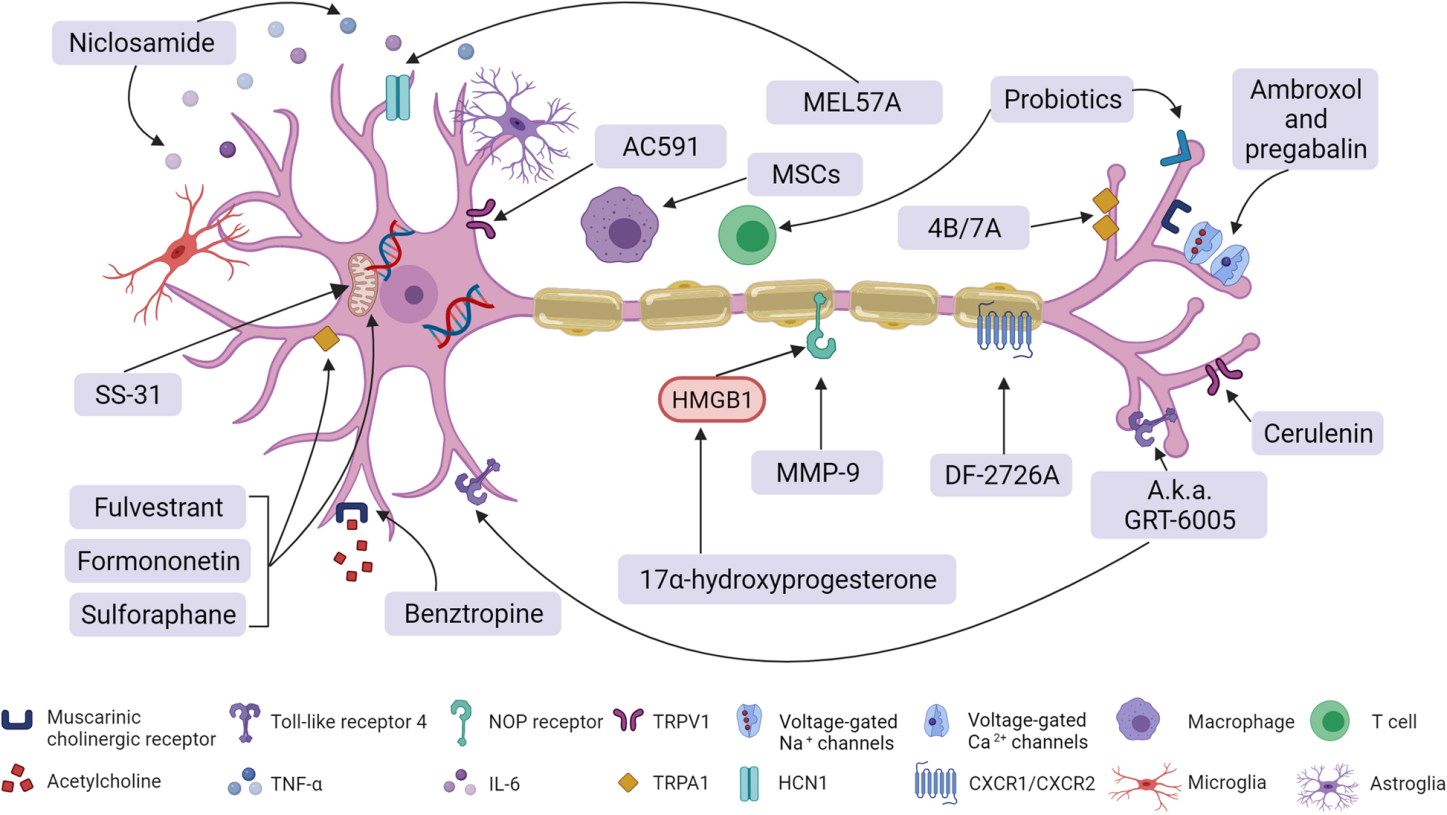
Potential therapies for CIPN caused by oxaliplatin. Repurposed drugs and preclinically tested lead compounds for OIPN

OIPN prevention and treatment still needs additional preclinical studies, which can provide useful information for innovative research and future trials. The anti-inflammatory therapies, antioxidant therapies, neuroprotective and anti-allodynia agents, combined therapies, OHP dosage regulation agents, and herbal medicinal therapies involving stem cells and gut microbiota (probiotics) are all subjects of promising ongoing research. For the anti-inflammatory therapies, the anti-macrophage-derived high mobility group box 1 (HMGB1) neutralizing antibodies therapy decreased the neuropathy of OIPN by neuroinflammation modulation in an OIPN mice model [161]. The dual inhibitor (compound-DF2726A) of chemokine receptor CXCR1/CXCR2 ligands was also studied in the OHP-induced neuropathy model [82]. Furthermore, the development of OHP-induced mechanical hypersensitivity was prevented by the administration of anti-CCL2 antibodies [121]. For the antioxidant therapies, our previous findings revealed that Nrf2 may have acted as a critical role in the progression of OIPN, with its ability of mitochondrial function protection and the inhibition of expression of the TRP protein family. The therapeutic or pharmacological activation of Nrf2 may be used to prevent or ameliorate the progression of OIPN without affecting the cytotoxicity of OHP [67, 80, 201]. Novel mitochondria-targeting antioxidants SS-31 prevented acute neuropathy symptoms caused by OHP in mice [164]. The neuroprotective agent niclosamide displayed potential antitumor and neuroprotective effects without affecting the cytotoxicity efficacy of OHP [202]. The 17α- hydroxyprogesterone caproate (HPGC), a synthetic derivative of progesterone, prevented the allodynia induced by OHP and glial activation in mice [201]. The anti-Parkinson’s agent benztropine, an anti-dopamine and histamine reuptake inhibitor, reduced OIPN severity in the OHP mouse model, synergizing its anti-tumoral effect [118]. Thrombomodulin α prevented OIPN induced allodynia, most likely via a thrombin-dependent anti-neuropathic action [37]. A novel hyperpolarization-activated cyclic nucleotide-gated channel 1(HCN1) inhibitor, MEL57A, recently demonstrated anti-hyperalgesic and anti-allodynic properties in OHP-treated rats [165]. The subcutaneously administered first-in-class potent analgesic compound cebranopadol (a.k.a. GRT-6005) reduced cold allodynia in the acute and the chronic phase of OIPN [166]. Mechanisms of CIPN overlap and can reinforce each other. The results of the hyperadditive effect with the combined subanalgesic doses of ambroxol and pregabalin indicated a synergistic reaction between the Na_v_ and Ca_v_ channel inhibitors in CIPN caused by OHP [64, 167]. The dual phosphodiesterase 4B/7A inhibitors and TRPA1 channel antagonists (HC-030031) reduced tactile allodynia in an OIPN model with TNFα-lowering effect [168]. Combined therapy using “traditional” pharmacological agents and mesenchymal stem cells (MSCs) may also be important in the prevention and treatment of CIPN [203]. Based on the double action of OHP dosage in neuropathy and chemotherapeutic effect, some chemosensitizers such as cerulenin may enhance the therapeutic effects of OHP, and thus lower the OHP-induced neuropathy [204]; however, individual precision treatment strategies warrant further exploration [205]. Gut microbiota (probiotics) have shown therapeutic effects in various diseases, and previous studies have also evaluated their protective role in various nociceptive pain states [103, 171]; however, at present, little is known about their potential role and effect on OIPN. Herbal medicinal therapies (e.g., AC591, *Acorus calamus*, *Camellia sinensi*, *Cannabis* species, *Curcuma longa*, *Ginkgo biloba*, and *Matricaria chamomilla*) counteract the phenomena underlying CIPN, i.e., they attenuate inflammation and reduce oxidative stress in animals [206, 207]; however, their utility in the prevention or treatment of CIPN requires further investigation to confirm their safety and efficacy [208, 209]. Preclinical studies regarding potential future preventive therapies for OIPN are presented in Fig. 4.

## Conclusion

Neuropathy is the most common side effect of oxaliplatin treatment and adversely affects patients’ daily life and chemotherapy progress. Regarding the various signs and symptoms from different OIPN patients, there is an urgent need to find methods for accurate diagnosis of the severity of neuropathy. Developing the use of neuroimaging and QST combined with AI may have great value in the future diagnosis and treatment of OIPN. It is important to identify biological effects and risk factors of OIPN to appropriately treat neuropathy. Although no clear OIPN clinical biomarkers can be used to measure OIPN vulnerability, a detailed phenotyping approach (including psychophysical testing, validated patient self-report questionnaires, neuroimaging, and genetic testing) may move toward simple bedside tests as surrogate markers and improve clinical trials for CIPN. Many reported mechanisms listed above can lead to oxaliplatin-induced neuropathy. However, progress in OIPN treatment and prevention has been limited by the superficial understanding of the OIPN mechanism. Mechanisms rely on the unique structure and function of the neurons, and the reticular interaction between neurons, the immune system, and cancer cells must be considered. OIPN treatment should be followed-up with the newly discovered mechanism.

An “ideal” OIPN agent should act as a multitarget agent to increase its neuropathy protective efficacy while not reducing the antitumor efficacy of the chemotherapy. Although there are some promising potential therapies for OIPN prevention and treatment, the need for improving the translation efficiency remains urgent. We need to draw lessons from previous failures in order to translate promising potential compounds into preclinical studies and early-phase clinical trials to expand clinical benefit. Moreover, the clinical trials also require design improvements, and careful attention should be paid to the clinical course and assessment methods of OIPN to gain useful and reliable information for prevention or treatment of severe side effects of OHP administration. Both the clinical evidence and preclinical studies need to include a critical appraisal of the quality and risk of bias. Issues such as blinding, prospectively defining the primary outcome measures, and proper sample size calculations should be considered [210]. Finally, identifying reliable biomarkers that can predict the OIPN clinical course will improve the clinical OIPN research toolkit. The relevance of the OIPN model to the clinical syndrome is also important, e.g., the behavioral assessment of spontaneous pain obtained by studying gender-specific rodents [211]. The prevention and treatment of OIPN remains an unmet and emergent clinical need. Further, to achieve effective and reliable results, high-quality research is mandatory.

In conclusion, OHP is a commonly used effective chemotherapy drug for digestive system tumors. Neuropathy is the most common side effect and exhibits dose-limiting and life quality affecting characteristics. The OIPN problem demands strong translational approaches to achieve successful clinical application. Well-designed preclinical studies are needed, reflecting the clinical situation and careful considerations of the clinical trials design. Working together to standardize assessment techniques and ensuring that those that are used are validated robustly is important. The potential OIPN mechanisms are abundant and thus offer promising targets for novel therapies. A holistic approach is required, beyond traditionally predominant pharmacological interventions, and needs consideration of mechanistically driven nonpharmacological interventions. Although OIPN is a challenge not to be underestimated, collaboration both between countries and disciplines will be the key to success, with many potential barriers and rewards.

## Data Availability

Not applicable.

## Abbreviations

ABCC2: ATP binding cassette subfamily C member 2
AGXT: Alanine glyoxylate aminotransfer-ase
AI: Artificial intelligence
Akt2: Protein kinase B
APE1: Apurinic/apyrimidinic endonuclease
ASCO: American Society of Clinical Oncology
ASCO: American Society of clinical Oncology
AT2: Angiotensin II type 2
ATP: Adenosine-triphosphate
ATP7A: ATPase Copper Transport-ing Alpha
BMI: Body mass index
Cavα2δ − 1: Calcium voltage-gated calcium channel alpha2/delta subunit
CCL2: C-C motif chemokine 2
CCR2: C-C-Motif Receptor 2
CD4 +: Cluster of Differentiation 4 receptors
CD8 +: Cluster of Differentiation 8 receptors
CIPN: Chemotherapy-induced peripheral neuropathy
CNS: Central nervous system
COX-2: Cyclooxygenase-2
CRC: Colorectal cancer
CTR1: Copper transport 1
CTRs: Copper transpo-rters
CXCR1: CXC chemokine receptor 1
CXCR2: CXC chemokine receptor 2
DDTC: Diethyldithiocarbamate
DNA: Deoxyribonucleic acid
DRG: Dorsal root ganglion
EAAT1: Recombinant Excitatory Amino Acid Transporter 1
EORTC: European Organization for Research and Treatment of Cancer
EPM: Electrophysiological measurements
ERK1/2: Extracellular regulated kinase1/2
GABA: γ-aminobutyric acid
GAT-1: GABA transporter subtype 1
GF: Germ-free
GFAP: Glial fibrillary acidic protein
GJG: Goshajinkigan
GLAST: Glutamate/aspart-ate transporter
GLT-1: Glutamate transporter-1
GM1: Monosialotetra-hexosylganglioside
GSTP1: Glutathione S-transferase pi 1
HCN1: Hyperpolarizationactivated cyclic nucleotide-gated channel 1
HCNs: Hyperpolarization-activated channels
HMGB1: High mobility group box 1
HPGC: Hydroxyprogesterone caproate
hTRPA1: Human TRPA1
IB: Investigator’s Brochure
Iba1: Ionized calcium binding adapter molecule 1
IENFs: Intraepidermal nerve fibers
IL-8: Interleukin-8
INCAT: Inflammatory Neuropathy Cause and Treatment
JNK/Sapk: c-JunNH2-terminal kinase /stress activated protein kinase
K2P: Two pore potassium channels
KCNN3: Potassium channel SK3
LDI: Laser Doppler imager
LPS: Lipopolysaccharides
MAMPs: Microbe-associated molecular patterns
mISS: Group sensory sum score
MMP9: Matrix metallopeptidase 9
MnSOD: Manganese superoxide dismutase
MSCs: Mesenchymal stem cells
NCI-CTC: National Cancer Institute-Common Toxicity Criteria
NGF: Mus musculus nerve growth factor
Nrf2: NF-E2 p45-related factor 2
OATP1B2: Organic aniontransporting polypeptide 1B2
OCT2: Recombinant Octamer Binding Transcription Factor 2
OCTN2: Organic cation transporter-2
OCTs: Organic cation transport-ters
OHP: Oxaliplatin
OIPN: Oxaliplatin-induced peripheral neurotoxicity
OXA: Oxaliplatin
PI3K: Phosphatidylinositide 3-kinases
PKC: Protein kinase C
PO: Per os
p38: Phosphoprotein 38
QST: Quantitative sensory tests
ROS: Reactive oxygen species
rhuLIF: Recombinant human leukemia inhibitory factor
rTMS: Repetitive transcranial magnetic stimulation
S1P: Sphingosine-1-phosphate
Sapk: Stress activated protein kinase
SK3: Small-conductance calcium-activated potassium channel 3
SNRI: Serotonin-noradrenaline reuptake inhibitor
SPF: Specified Pathogen Free
S1P2: Sphingosine-1-phosphate receptor2
S1PR1: Sphingosine-1-Phosphate Receptor 1
TLR: Toll-like receptors
TLR4: Toll-like receptors 4
TNSc: Total Neuropathy Score clinical version
TRAAK: TWIK-related arachidonic acid-stimulated K+ channel
TREK-1: TWIK-Related K + Channel 1
TRPA1: TRP ankyrin 1
TRPM8: Transient receptor potential cation channel, subfamily M, member 8
